# Uptake of modern contraceptive methods among women of reproductive age in Chake District-Pemba Tanzania: a descriptive crossectional study

**DOI:** 10.1186/s40834-023-00234-y

**Published:** 2023-07-17

**Authors:** Rehema Abdalla Abeid, Emmanuel Izack Sumari, Chunxiang Qin, Ally Abdul Lyimo, Godrian Aron Luttaay

**Affiliations:** 1 Department of Preventive Services, Integrated Reproductive and Child Health Program, Ministry Health, Pemba, Tanzania; 2 Department of Nursing Management Muhimbili University of Health and Allied Sciences, Dar es Salaam, Tanzania; 3 Department of Nursing, The Third Xiangya Hospital, Central South University, Changsha, 410013 China; 4 Department of Community Nursing, Muhimbili University of Health and Allied Sciences, Dar es Salaam, Tanzania; 5 Department of Nursing, Kibosho Institute of Health and Allied Sciences, Moshi, Tanzania

**Keywords:** Modern contraceptives, Family planning, Uptake, Factors, Barriers

## Abstract

**Background:**

The uptake of the modern contraceptive method provides chances for women and couples to reach optimal child spacing, achieve the desired family size and prevent unsafe abortions and maternal deaths. Despite the efforts in the health sector still, the contraceptive prevalence rate in Zanzibar remains low (9.1%). In Pemba, few studies have been done on modern contraceptive uptake and little is known about factors that hinder the uptake of modern contraceptives among women of reproductive age. This study investigated the uptake of modern contraceptive methods among women of reproductive age (18-45 years) and its associated factors.

**Methods:**

This was a quantitative cross-sectional study conducted in Chake District Hospital, Pemba Tanzania. A stratified random sampling technique was used to recruit 214 eligible participants for the study. After we informed the participants, data were collected using a structured English questionnaire. The collected data was analyzed using SPSS version 25, descriptive analysis was done to determine frequencies. A chi-square test was done to determine the association between the study variables and multivariate logistic regression to check the nature and strength of the association. The *p<0.05* was considered statistically significant.

**Results:**

This study included 214 women of with majority 79(36.9%) at the age group of 21-30 years, 100(46.7%) had secondary education and 187(87.4%) married. Most of the participants 212(99.1%) have heard about modern contraceptives, with health facilities being the common source of information191(45.3). More than half 120(56.1%) of the participants were not using any modern contraceptive method and injectable 38(40.4%) was the commonly reported method among users. Among the users of modern contraceptives, lack of power to decide 180(84.1%), fear of divorce 141(65.9%), and social perception of users as the cause of reduced workforce in the future 161(75.2%) were common barriers. Participants provided suggestions to improve modern contraceptive uptake including male involvement 203(94.9%) and community awareness 182(85%). Further analysis revealed women with college/university education were 2 times more likely to use modern contraceptives method compared to those with primary or not attended school(*p=0.023*, OR=2.437, 95% CI: 1.129-5.259). Moreover employed women were 2 times more likely to use modern contraceptives compared to unemployed/housewives (*p=0.028*, OR=1.844, CI=1.068-3.185).

**Conclusion:**

This study assesses the uptake of modern contraceptives among women of reproductive age. Results showed a low uptake of modern contraceptives in this population.

Although the observation in this study is similar to those reported in other countries, the updated information is still important to the policymakers and the Ministry of Health in the studied district.

## Introduction

### Background

The uptake of modern contraceptives (pills, injectables, intra- uterine contraceptive device, implant, condom, female sterilization, male sterilization) remains a public health concern in most countries. In 2019 among 1.9 billion women of reproductive age, 1.1 billion needed family planning, out of these 842 million used modern contraceptives and 270 million had unmet needs for modern contraceptives [1, 2]. Global estimates for the need for family planning suggest an increase to 1.19 billion women by 2030 and 918 million users of modern contraceptives, with more than half from low and middle-income countries [1]. Most of the countries with women of reproductive age in need of preventing pregnancy but not using any modern contraceptive are from Sub-Sahara Africa [3]. Modern contraceptive use rate for women aged 15-49 is twice high among women living in high-income countries compared to low-income countries 67%, and 34% respectively [4, 5].

In sub-Saharan Africa fertility rate is high while modern contraceptive uptake is low; every year there are 14 million unwanted pregnancies and 25% of women of reproductive age do not meet the need for modern contraceptive use [6, 7]. In Tanzania, the prevalence of modern contraceptive use among women of reproductive age (15-45) is 32% [7, 8]. In Zanzibar, although the government provides free modern contraceptive methods both in Unguja and Pemba, its prevalence rate remains low (28%) [5, 9]. In 2016, in Zanzibar, there was a minor decrease in the unmet need for contraception to 28% but of all the demand modern contraceptive was supposed to cover 27.3% [10]. Modern contraceptive uptake in Pemba is (9.1%) which is even very low than in any other region of Tanzania [5].

The use of modern contraceptives has been reported to have several benefits such as; prevention of unwanted pregnancy, ensuring proper family size and children at planned intervals, giving lactation mother women enough time to recover, taking care of the new-born and allowing them to participate in other social and economic activities [11]. Moreover, modern contraceptive use has been useful in the prevention of sexually transmitted diseases and HIV/AIDs, ensuring steady population growth which reduces competition on the available resources [12, 13].

Previous studies reported that the unmet need for modern contraceptive use may be associated with several outcomes including increased risk of pregnancy-related problems, increasing unsafe abortion, poverty and diminished economic security for the communities and households, and increased maternal deaths [5, 14]. In Tanzania, maternal mortality rate (MMR) infant mortality rate (IMR), and neonatal mortality rate (NMR) is 556 death/100,000 live births, 43 deaths/1000 live births, and 25 deaths/1000 live births respectively [5, 14, 15]. Deaths of women of reproductive age who are considered an economically productive group are associated with economic decline at the family and national level [16].

In Tanzania studies have been done to determine the prevalence and factors associated with modern contraceptive use, however, the focus has been on urban areas. In Zanzibar, especially Pemba few studies have been done on modern contraceptive use [17, 18]. This study assessed the uptake of modern contraceptives and their associated factors among women of reproductive age (18- 45) in Chake District, Pemba.

## Methodology

### Aim of the study

This study aims to assess the uptake of modern contraceptives and their associated factors among women of reproductive age (18- 45).

### Study design

The study used a quantitative cross-sectional design to assess the uptake of modern contraceptives, data collected from October to December 2020.

### Study setting

This study was conducted in Chake District Hospital, Pemba Tanzania. Chake District Hospital is a public institution located in the urban area of the island that provides services to the community around and receives referrals from lower-level health facilities. The hospital has several departments such as obstetrics and gynecology, general inpatient and outpatient medical and surgical services, and reproductive and child health clinic (RCH). The RCH provides antenatal care (ANC) and postnatal services on weekdays without payment. The clinic provides services to about 50 women who come to the RCH clinic daily and approximately 1000 each month.

### Study population

This study targeted women of reproductive age (18-45) years accessing RCH services at Chake District Hospital.

#### Sample size

This study included a total sample of 214 participants which was calculated using Cochran's formula [19].

$$\mathrm{n }= \frac{{\mathrm{Z}}^{2}\mathrm{ pq}}{{\mathrm{e}}^{2}}$$

Where n is the sample size, z is the z – score = 1.96 on the normal standard variable curve corresponding to 95% confidence level, e is the desired level of precision, e=0.05 and q =1 – p where p = proportion of the population with the characteristic of interest = 14.3%. This p-value was adopted from a study done in the Democratic Republic of Congo which reported that only 14.3% of the population used modern contraceptive methods [20].

$$\mathrm{n}= \frac{1.9{6}^{2}\mathrm{ x }0.143\mathrm{ x }0.857}{0.0{5}^{2}}$$

$$\mathrm{n}= 188$$

Therefore, the final sample size for this study was 188 + 10% of the sample for incomplete questionnaires and non-response = 207 participants.

#### Sampling technique

This study used a stratified random sampling technique to recruit 214 participants. Women coming for RCH services met in two points which were the identified strata; Strata 1 included women accessing ANC services (family planning, prevention of mother to child transmission (PMTCT), screening for cervical cancer, growth monitoring and plotting (GMP); Strata 2 included women accessing post-natal services (post-natal care, immunization for under five, sick children and management of women with gynecological problems). Each stratum contributed a minimum of 50% of the required sample. From the specific clinics approximately 27 participants were randomly selected in each stratum until the final number was achieved.

#### Inclusion and exclusion criteria

The study included women of reproductive age (18-45 years) attending the RCH clinic in Chake Hospital while women with serious mental or medical illnesses or taking care of very sick children were excluded.

### Research tool

Data for this study were collected using a structured English/Swahili translated questionnaire. The questionnaire was developed following a thorough review of published studies on barriers and factors associated with modern contraceptive uptake conducted in different countries [13, 21–28]. The questionnaire comprised three sections with multiple choices; Section A: demographic information; Section B: Questions on modern contraceptive uptake and its associated factors; Section C: other influencing factors and suggestions to improve modern contraceptive uptake.

### Validity and reliability

The developed questionnaire was shared with experts to check if the questions answers the research objectives/content validity. No major comments were identified rather arrangement and grammatical suggestions were. All suggestions were addressed and before the actual data collection, the research tool was pretested on 20 participants to check for reliability. Participants were able to complete the questionnaire in 10 minutes and no concerns were raised about the tool. The collected information was entered on SPSS to determine the reliability of the tool by using a Likert-rating scale that revealed acceptable results (Cronbach alpha =0.78). The research questionnaire was available in English and Swahili versions for easy use by the study participants.

### Data collection technique

Data for this study was collected by self-administration of the questionnaire and for a few women who could not read or write we interviewed them and assisted in filling in their answers. Data from the two strata ANC and postnatal were collected on alternating days. We met the participants individually as they were waiting for RCH services, explained the study, its aim, and right to participation, and informed them that their denial to participate could not affect their right to treatment. Each participant was given two copies of consent forms and after signing, they filled out the questionnaire and submitted it immediately.

### Statistical analysis

The data collected was entered, cleaned, and analyzed by using SPSS for Windows version 25.0. Descriptive analysis was done to determine the frequencies and percentages for demographic characteristics and information on modern contraceptive uptake, cultural and other factors. A chi-square test was done to determine the association between the participant's characteristics and modern contraceptive uptake status, *p*<0.05 was considered statistically significant. Multivariate regression analysis was done to determine the nature of the association between the participant's characteristics and modern contraceptive uptake.

## Results

### Sociodemographic characteristics of the participants

The study included 214 women and half of them had an age range of 21-30 years 107 (50%). Most of them 195 (91.1%) were Muslim and 100 (46.7%) had secondary education. The majority of the participants187 (87.4%) were married and 107 (50%) were employed (self-employed, civil servant, private) (Table 1).

**Table 1:** Sociodemographic characteristics of the study participants

Demographic characteristic	Frequency (N)	Percentage (N %)
**Age Group**		
< or equal 20	11	5.1
21-30	107	50
31-40	79	36.9
> or equal 41	17	7.9
**Religion**		
Muslim	195	91.1
Christian	18	8.4
No religion	1	0.5
**Education level**		
Primary/below	68	31.8
Secondary	100	46.7
College/ university	46	21.5
**Occupation**		
Employed (Civil servant, private and self-employed)	107	50
Unemployed (Housewife)	107	50
**Marital status**		
With a partner (Married)	187	87.4
With no partner (single, divorced, widow)	27	12.6

### Information about modern contraceptive uptake

In this study, we found that 212(99.1%) women have heard about modern contraceptives with health facilities being the main source of information 191 (45.3%). More than half of women 120(56.1%) were not users of modern contraceptives currently while injectable was the common method among the users 38 (40.4%) (Table 2).

**Table 2:** Information about modern contraceptive uptake

Variable	Frequency (N)	Percentage (N %)
**Ever heard about modern contraceptives**		
Yes	212	99.1
No	2	0.9
**Source of information on modern contraceptives**		
Health facility	191	45.3
Friends	89	21.1
Media TV/radio	75	17.8
School	25	5.9
Internet	37	8.8
Other (Mother, newspaper, poster)	5	1.2
**Currently using modern contraceptive**		
Yes	94	43.9
No	120	56.1
**The family planning method currently using**		
Pills	13	13.8
Injectable	38	40.4
IUCD	8	8.5
Implant	23	24.5
Condom	6	6.4
Male sterilization	1	1.1
Calendar	5	5.3

### Barriers for modern contraceptive uptake and social perception

Among the cultural barriers, the lack of power to decide on modern contraceptive use was commonly mentioned in 180 (84.1%). Women using modern contraceptives were perceived as the reason for reduced manpower in the future 161 (75.2%) and fear of divorce was identified by the majority 141 (65.9%) as another barrier affecting modern contraceptive uptake (Table 3).

**Table 3:** Barriers for modern contraceptive uptake and social perception

Variable	Frequency (N)	Percentage (N %)
**Cultural barriers associated with the modern contraceptive uptake**		
Against my Religion	114	53.3
Against value and norms	144	67.3
Bad practice	24	11.2
Community stigmatization	160	74.8
No power to decide	180	84.1
Need more children	163	76.2
No support from husband	155	72.4
**Social perceptions on modern contraceptive uptake**		
Engage in the extra sexual act	27	12.6
Modernized	147	68.7
Evil	30	14.0
Reduction of manpower in future	161	75.2
Good practice	71	33.2
**Other barriers associated with the modern contraceptives**		
Fear of side effects	127	59.3
Modern contraceptives stoke out	13	6.1
Distance	16	7.5
Staff competence	6	2.8
Fear of divorce	141	65.9
Poor awareness/ knowledge	7	3.3

### Information received and suggestions to improve modern contraceptives uptake

In this study, participants said that the common information provided by the health care providers was showing different methods 202 (94.4%). Moreover, to improve modern contraceptive uptake, male involvement 203 (94.9%) was the main identified suggestion (Table 4).

**Table 4:** Information received and suggestions to improve modern contraceptives uptake

Variable	Frequency (N)	Percentage (N %)
**Information is given about modern contraceptives**		
Show different modern contraceptive methods	202	94.4
Explain how it works	190	88.8
Explain the side effects	185	86.4
Explain actions to take	82	38.3
**Suggestions to improve modern contraceptive uptake**		
Male involvement	203	94.9
Community engagement for awareness	182	85.0
Public campaign	157	73.4
Strengthen the quality of modern contraceptive	152	71.0
Strengthening structural factors	143	66.8

### Association between participants’ characteristics and modern contraceptive uptake

It was found that the education level and occupation of the participants had a significant association with modern contraceptive uptake *p=0.011* and *p=0.028* consecutively (Table 5). Moreover, multivariate logistic regression for the factors which were found to have significant association revealed that women with college/university education were 2 times more likely to use modern contraceptives than those with primary or not attended school (*p=0.023*, OR=2.437, CI=1.129-5.259). Furthermore, employed women were about 2 times more likely to to use modern contraceptives than unemployed/housewives (*p=0.028*, OR=1.844, CI=1.068-3.185) (Table 6).

**Table 5:** Association between participants’ characteristics and modern contraceptive uptake

	Using modern contraceptive			
Variable	Yes (%)	No (%)	χ 2	*p*-value
**Age group**			1.562	0.668
< or equal 20	3 (27.3)	8 (72.7)		
21-30	47 (43.9)	60 (56.1)		
31-40	37 (46.8)	42 (53.2)		
> or equal to 41	7 (41.2)	10 (58.8)		
**Religion**			1.829	0.401
Muslim	84 (43.1)	111 (56.9)		
Christian	10 (55.6)	8 (44.4)		
No religion	0 (0)	1 (100)		
**Education level**			8.982	***0.011***
Primary/below	28 (41.2)	40 (58.8)		
Secondary	37 (37)	63 (63)		
college/ university	29 (63)	17 (37)		
**Marital status**			0.788	0.375
With a partner/married	80 (42.8)	107 (57.2)		
No partner (single, widow, divorced)	14 (51.9)	13 (48.1)		
**Occupation**			4.857	***0.028***
Employed (self, civil servant, private)	55 (51.4)	52 (48.6)		
Not employed (Housewife)	39 (36.4)	68 (63.6)		
**Fear of side effects**			0.251	0.617
Yes	54 (42.5)	73 (57.5)		
No	40 (46)	47 (54)		
**Modern contraceptives stoke out**			0.553	0.457
Yes	7 (50)	7 (50)		
No	87 (43.3)	114 (56.7)		
**Distance**			1.066	0.302
Yes	7 (43.8)	9 (56.2)		
No	85 (42.9)	113 (57.1)		
**Staff competence**			3.892	0.089
Yes	5 (83.3)	1 (16.7)		
No	89 (42.8)	119 (57.2)		
**Fear of divorce**			0.36	0.548
Yes	64 (45.4)	77 (54.6)		
No	30 (41.1)	43 (558.8)		
**Poor awareness/ knowledge**			0.513	0.474
Yes	4 (57.1)	3 (42.9)		
No	90 (43.5)	117 (56.5)

**Table 6:** Multivariate logistic regression of the predictors of modern contraceptive uptake among participants

Variable	B	S.E.	Wald	df	p-value	OR	95% CI
**Education**							
Primary/below (ref)	-	-	-	-	-	1	-
Secondary	-0.176	0.322	0.297	1	0.586	0.839	0.446-1.577
College/university	0.891	0.392	5.152	1	***0.023***	2.437	1.129-5.259
**Occupation**							
Employed	0.612	0.279	4.817	1	***0.028***	1.844	1.068-3.185
Unemployed (ref)	-	-	-	-	-	1	-

## Discussion

In this study, the majority of women have heard about modern contraceptive methods and the main source of information was health facilities. A different study done in Rwanda reported that only 47% have heard about modern contraceptives [27]. Other studies reported different results where about 85% and 57% of the participants had information from radio [26, 29]. More studies also showed that friends/peers and family were the main source of information [30–32]. The findings in the current study may be explained by the measures that have been in place and future targets by the government through the Ministry of Health ensuring that modern contraceptive services are provided to all levels of facility [33].

In the present study, although most women have heard about modern contraceptives, the majority were currently not using any. This is similar to studies from Pakistan, Saudia, and Ajman which showed that about 75%, 55%, and 61% respectively never used any modern contraceptive methods [31, 32, 34]. Additionally, another study reported that compared to Christians, Muslims were 65% less likely to uptake modern contraceptives although they were informed and knew where to access the services [35]. Contrary, a study which was done in Oman showed that 54% of women used modern contraceptives [36]. These differences may be influenced by how families believe and practice their culture and religion as explained in a study which reported that those who find contraceptives contradicting with religion and culture give birth to children as God gives [37].

Moreover, among the users of modern contraceptives, injectables and implants were the common methods similar to a previous study where users preferred the same methods [25]. Other different methods were reported in previous studies where about 78% and 31% of the participants used condoms as a common method [32, 38]. A study which was done in Saudia revealed that pills were the common method among users 71% [39]. The findings in the present study may be because injectables might be more convenient and friendly in terms of privacy since some women do not want their husbands to know if they use modern contraceptive [38].

The present study also identified social and cultural factors as barriers to modern contraceptive uptake. Participants said that its use is against religious values and norms, accompanied with community stigmatization, the lack of power to decide to use, the need for more children, and lack of support from husband. Studies done in Oman, Ethiopia, and Pakistan also reported similar factors; need for more children 43% and 38%, 50% spouse opposition respectively [34, 36, 40]. More studies reported similar information where about 90% of the participants experienced opposition from their husbands, community stigma 55% [41, 42], need for more children by husband [43], and influence of religion [23, 32]. Contrary, two previous studies reported that about 91% and 68.5% of the women respectively had full support from their husbands on the use of modern contraceptives and support in buying and transportation [36, 44]. As for this study and those done in countries with similar cultures and religious beliefs, men have the authority and power to decide on issues related to the health of the family, if they are not ready most women will respect them because doing otherwise is perceived a disrespect [45, 46] and may lead to serious consequences including divorce.

Furthermore, it was found that women with college/university education were 2 times more likely to uptake modern contraceptives compared to those with primary or not attended school similar to findings in other previous studies [7, 25, 27, 28, 36]. In addition, the present study revealed that employed women were 2 times more likely to use modern contraceptives compared to unemployed/housewives similar to other previous studies [20, 23, 28]. The findings in the present study may be because reproductive health education and modern contraceptive information have now extended and it’s provided in schools, colleges, universities, health facilities, and media. In addition, most educated women are either employed or self-employed hence they may prefer modern contraceptive use so that they can have enough time to participate in different productive activities.

Moreover, in this study, modern contraceptive use was reported to have different community perceptions. The participants said that users of modern contraceptives were perceived as modernized. This is different from a previous study where users were considered to have bad behavior and informed consent to use from the husband was mandatory 75% [39]. Also participants said that modern contraceptive use will result in reduction of manpower in future similar to a study which reported that, God’s plan is that marriage should be fruitful and the church does not support modern contraceptive use [47]. These perceptions may be due to awareness, cultural background, and religious beliefs that can affect modern contraceptive uptake [48].

In the present study, participants had different suggestions to improve the uptake of modern contraceptives including; male involvement, community engagement for awareness, and public campaigns. Community and male partner involvement were also recommended in the previous study due to their influence on women’s decision to use modern contraceptives, including to use or discontinuation [45]. The suggestions in this study could be because women feel that male and community involvement have not been well addressed as other barriers like availability, accessibility, distribution for free, and training of experts.

### Limitations of the study

This study used a stratified random sampling technique to obtain the participants; selecting participants randomly from the strata might result in selection bias but we considered different participants’ characteristics to get representativeness.

## Conclusion

The finding of the present study provides a similar picture to previous studies done in settings with similar social and cultural backgrounds. However this findings provides a useful and updated information on modern contraceptive use to the important stakeholders in the studied district. The findings calls for more interventions from the Ministry of Health and other key stakeholders to plan different programs targeting male involvement, community engagement for awareness, and strengthening the quality of modern contraceptive services.

## Data Availability

When necessary, the research tools, dataset, and other materials supporting the results will be shared upon consultation with the corresponding author.
